# Ethylene-independent promotion of photomorphogenesis in the dark by cytokinin requires COP1 and the CDD complex

**DOI:** 10.1093/jxb/ery344

**Published:** 2018-09-29

**Authors:** Anne Cortleven, Stephanie Ehret, Thomas Schmülling, Henrik Johansson

**Affiliations:** Institute of Biology/Applied Genetics, Dahlem Centre of Plant Sciences, Freie Universität Berlin, Berlin, Germany

**Keywords:** *Arabidopsis thaliana*, cytokinin, ethylene, photomorphogenesis, light signaling, two-component signaling system

## Abstract

The transition of skotomorphogenesis to photomorphogenesis is induced by the perception of light, and is characterized by the inhibition of hypocotyl elongation and opening of cotyledons. Although it is known that the plant hormone cytokinin inhibits hypocotyl elongation in dark-grown Arabidopsis plants when applied in high concentrations, it is unclear to what extent this response is the result of cytokinin alone or cytokinin-induced ethylene production. Here, we show that cytokinin-induced inhibition of hypocotyl elongation is largely independent of ethylene and suggest a close connection between the cytokinin two-component system and the light-signaling networks. We show that this cytokinin signal is mainly mediated through the cytokinin receptor ARABIDOPSIS HISTIDINE KINASE3 and the ARABIDOPSIS RESPONSE REGULATOR1 in combination with ARR12. Interestingly, mutation of *CONSTITUTIVELY PHOTOMORPOGENIC1* (*COP1*), *DE-ETIOLATED1*, and *CYTOKININ INSENSITIVE4*/*COP10* renders plants insensitive to cytokinin, and these factors are indispensable for the transcriptional response during cytokinin-induced de-etiolation, indicating that a functional light-signaling pathway is essential for this cytokinin response. In addition, the effect of cytokinin on hypocotyl elongation is strongly dependent on the light conditions, with higher light intensities causing a switch in the response to cytokinin from an inhibitor to a promoter of hypocotyl elongation.

## Teaser

Cytokinin promotes photomorphogenesis independent of ethylene, acting through the two-component signaling system and requiring a functional light-signaling system.

## Introduction

A seedling’s transition from skotomorphogenic to photomorphogenic growth (de-etiolation) after exposure to light marks the transition from heterotrophic to photoautotrophic growth. This developmental switch, which is driven by genome-wide transcriptional reprogramming, results in dramatic phenotypic changes, including unfolding of the apical hook, rapid inhibition of hypocotyl elongation, opening and expansion of the cotyledons, and the promotion of chloroplast development (Ma *et al.*, 2001; Sullivan and Deng, 2003).

In Arabidopsis, four major classes of photoreceptors perceive incoming light and regulate de-etiolation; these sense UV-B (UVR8 receptor) (Rizzini *et al.*, 2011), blue (cryptochromes and phototropins) (Briggs and Christie, 2002; Yu *et al.*, 2010), and red/far-red (phytochromes) (Wang and Deng, 2004) light. In addition to these photoreceptors, a number of positive and negative regulators of photomorphogenesis have been identified in a series of genetic screens over the past decades. *COP1* (*CONSTITUTIVELY PHOTOMORPOGENIC1*), *DET1* (*DE-ETIOLATED1*), and *FUS* (*FUSCA*) were identified as negative regulators of light signaling owing to the photomorphogenic phenotype of their respective mutants in darkness (for a review, see Kim *et al.*, 2002). COP1 directly associates with SPAs (SUPPRESSOR OF PHYA) to act as a substrate receptor of a CUL4-DDB1 (CULLIN4-DAMAGED DNA BINDING PROTEIN1)-based E3 ubiquitin ligase complex that targets photomorphogenesis-promoting transcription factors such as ELONGATED HYPOCOTYL5 (HY5) for degradation (Chen *et al.*, 2010). DET1, CIN4/COP10, and DDB1 form a separate complex (CDD) that acts through CUL4 to promote the action of the COP1-SPA-DDB1-CUL4 E3 ubiquitin ligase (Yanagawa *et al.*, 2004; Chen *et al.*, 2006). Light signals perceived by the photoreceptors repress the activity of COP/DET/FUS, leading to the accumulation of transcription factors promoting photomorphogenesis (Lau and Deng, 2012). As well as the COP/DET/FUS proteins, PHYTOCHROME INTERACTING FACTORs (PIFs) are also essential for the repression of photomorphogenesis in darkness (for a review, see Leivar and Monte, 2014).

Given the importance of light throughout the plant life cycle (Sullivan and Deng, 2003) it is not surprising that the light-signaling networks are closely interconnected with several phytohormones (for a review, see Lau and Deng, 2010; Cortleven and Schmülling, 2015). Treatment with inhibitors of both brassinosteroid and gibberellin signaling (Li *et al.*, 1996; Alabadí *et al.*, 2004) and the application of either strigolactone (Tsuchiya *et al.*, 2010), jasmonic acid (Zheng *et al.*, 2017), or cytokinin (CK) (Chory *et al.*, 1994) have all been shown to inhibit skotomorphogenesis, resulting in seedling de-etiolation in the dark.

CK regulates numerous developmental processes such as the cell cycle, shoot and root meristem size and activity, and leaf senescence (for a review, see Werner and Schmülling, 2009). The CK signal is perceived by three membrane-localized sensor histidine kinases, ARABIDOPSIS HISTIDINE KINASE2 (AHK2), AHK3, and CYTOKININ RESPONSE1/AHK4 (CRE1/AHK4), which are predominantly located in the endoplasmic reticulum and have both distinct and overlapping functions (Inoue *et al.*, 2001; Suzuki *et al.*, 2001; Higuchi *et al.*, 2004; Nishimura *et al.*, 2004; Riefler *et al.*, 2006; Wulfetange *et al.*, 2011; for a review, see Heyl *et al.*, 2012). The CK signal is further transmitted by a two-component signaling system via HISTIDINE PHOSPHOTRANSFER PROTEINS (AHPs) to B-type RESPONSE REGULATORS (B-type ARRs), which are transcription factors that regulate CK-responsive genes (Sakai *et al.*, 2001; Argyros *et al.*, 2008).

The link between photomorphogenesis and CK was first made by Chory *et al.* (1994), who showed that Arabidopsis seedlings that germinated in the dark on CK-containing medium had a phenotype resembling that of *cop*/*det* mutants. Another mutant with increased endogenous CK levels, *amp1*, also shows a de-etiolated phenotype in darkness (Chin-Atkins *et al.*, 1996). Further, the *cin4*/*cop10* mutation renders Arabidopsis insensitive to the induction of the ethylene-mediated triple response by CK in the dark (Vogel *et al.*, 1998); this observation links CK action to light signaling, as COP10 has a central role in repressing photomorphogenesis (Lau and Deng, 2012).It has been suggested that the interaction between light and CK is mediated by HY5, a transcription factor that acts downstream of cryptochrome and phytochrome (Vandenbussche *et al.*, 2007).

Although the reports reviewed above all support a role for CK as a positive regulator of photomorphogenesis, the inhibition of hypocotyl elongation caused by CK in darkness has been attributed to increased ethylene signaling (Cary *et al.*, 1995). Ethylene induces the triple response in dark-grown seedlings; this response is characterized by a strongly curved apical hook, shortening of the roots, and shortening and thickening of the hypocotyl (Guzmán and Ecker, 1990; Wang *et al.*, 2002). Being a derivative of methionine, ethylene is produced in three steps involving *S*-adenylmethionine synthetase, 1-aminocyclopropane-1-carboxylic acid (ACC) synthase, and ACC oxidase (Yang and Hoffman, 1984). Furthermore, its biosynthesis is induced by CK (Woeste *et al.*, 1999), which increases the stability of ACC synthase (Hansen *et al.*, 2009), and this response is mediated through CRE1/AHK4 and ARR1. Owing to this CK-induced ethylene production, CK is thought to exert part of its influence on hypocotyl elongation through ethylene. In fact, the inhibitory effects of 6-benzylaminopurine (BA) on root and hypocotyl elongation were shown to be partially blocked by the action of ethylene inhibitors or ethylene-resistant mutations (Cary *et al.*, 1995). Besides ethylene biosynthesis, there is also a close correlation between CK and ethylene signaling. In particular, *arr2* mutant seedlings are hyposensitive to ethylene, comparable to *ein3* mutants, in a hypocotyl assay, suggesting that ARR2 is a signaling component functioning downstream of ETR1 in ethylene signal transduction (Hass *et al.*, 2004). All these reports support the notion that CK induces ethylene production and suggest that CK might exert part of its influence in regulating hypocotyl elongation by stimulating the ethylene pathway.

In this study, we re-examined the effect of CK on dark-grown seedlings and found that the photomorphogenic effect of CK is largely independent of ethylene. Treatment of etiolated seedlings with CK in the presence of ethylene inhibitors (e.g. AgNO_3_) clearly resulted in the inhibition of hypocotyl elongation. To investigate the ethylene-independent transcriptional response to CK in more detail, RNA-Seq analysis was performed and revealed that CK treatment causes a similar transcriptional response to light. Moreover, we showed that this CK response is mediated through AHK3 and the B-type ARRs ARR1 and ARR12. In addition, we explored the relationship between the known light-signaling networks and CK treatment, and found that the *cop1*, *det1*, and *cin4* mutants are insensitive to CK-induced hypocotyl inhibition in the dark and that these components of the light-signaling pathway are necessary for the transcriptional response to CK. With this work we provide a molecular framework for the ethylene-independent CK-induced inhibition of hypocotyl elongation, linking this CK response to the major negative regulators of photomorphogenesis.

## Materials and methods

### Plant material, growth, and treatment conditions

*Arabidopsis thaliana* Col-0 was used as the wild type (WT). The CK receptor mutants *ahk2-5*, *ahk3-7*, *cre1-2*, *ahk2-5 ahk3-7, cre1-2 ahk2-5*, and *cre1-2 ahk3-7* (Riefler *et al.*, 2006), B-type *arr* mutants *arr1-4*, *arr10-5*, *arr12-1*, *arr1-3 arr10-5*, *arr1-3 arr12-1*, and *arr10-5 arr12-1* (Mason *et al.*, 2005; Ishida *et al.*, 2008), ethylene-insensitive mutant *ein2-1* (Guzmán and Ecker, 1990), and light-signaling mutants *cop1-4*, *cop1-6* (McNellis *et al.*, 1994), *det1-1* (Chory *et al.*, 1994), *cin4* (Vogel *et al.*, 1998), and *hy5-215* (Oyama *et al.*, 1997) have been described previously. The *ahk2-5 ahk3-7 ein2-1* and *arr1 arr12 ein2-1* triple mutants were obtained by genetic crosses of *ein2-1* to *ahk2-5 ahk3-7* and *arr1 arr12*, respectively, and *cop1-4 hy5-215* was obtained by crossing *cop1-4* with *hy5-215*. Seeds were surface sterilized, sown on 0.5 × Murashige and Skoog (MS) medium (Murashige and Skoog, 1962) (1% agar) without sucrose, and stratified in darkness for 3 days at 4 °C before given a 2 h pulse of white light (75 µmol m^−2^ s^−1^). The 0.5 × MS medium was supplemented with 3 µM BA and/or 10 µM AgNO_3_ unless stated otherwise. Seedlings were grown in complete darkness in a growth cabinet (Percival AR66L; Percival Scientific, Perry, IA, USA) at 22 °C.

### Hypocotyl length and cotyledon size measurements

For experiments in darkness, hypocotyl length and cotyledon size measurements of 5-day-old seedlings grown on vertical plates were made using ImageJ Software (https://imagej.nih.gov/ij/). For experiments in light, seedlings were grown for 5 days under long-day (16 h light/8 h dark) conditions under white light (50 µmol m^−2^ s^−1^) before the length of the hypocotyl was measured. Experiments were performed at least twice.

### Analysis of transcript levels by RNA-Seq and real-time qPCR

Total RNA was extracted from 4-day-old etiolated seedlings grown on medium containing 10 µM AgNO_3_ in the presence or absence of 3 µM BA using the RNeasy Plant Mini Kit (Qiagen, Hilden, Germany) as described in the kit’s user manual, including an on-column DNAse treatment.

For RNA-Seq analysis, RNA was isolated from three biological replicates for each treatment. The isolated RNA was sent to BGI (Hong Kong, China) for RNA quality and integrity control, library synthesis, high-throughput sequencing, and bioinformatics analysis. In brief, the RNA concentration and integrity. and extent of rRNA contamination were monitored by the Nanodrop NA-1000 and the Bioanalyzer Agilent 2100 (Agilent Technologies, Santa Clara, CA, USA). After DNAse I treatment, mRNA was enriched by using oligo(dT) magnetic beads and fragmented into shorter fragments. First-strand cDNA was synthesized by using random hexamer primers, followed by second strand synthesis. After purification, end repair, and 3ʹ end single-nucleotide A (adenine) addition, sequence adaptors were ligated. Following PCR amplification and quality control by the Agilent 2100 Bioanalyzer and ABI StepOnePlus Real-Time PCR System (Thermo Fischer Scientific, Waltham, MA, USA), the library products were sequenced via the Illumina HiSeq^TM^4000 platform. More than 22 million raw sequencing reads were obtained for each sample. After the removal of adaptors and low-quality reads, the obtained clean reads (approximately 21 million) were stored in FASTQ format (Cock *et al.*, 2010). Sequences were aligned to the TAIR10 Arabidopsis reference genome using Bowtie2 (Langmead *et al.*, 2009). Gene expression levels were quantified using RSEM (Li and Dewey, 2011) and differentially expressed genes (DEGs) were analyzed using the NOISeq method (Tarazona *et al.*, 2011) with the following default criteria: fold change ≥2 and divergence probability ≥0.8. Gene Ontology (GO) annotation was performed using DAVID 6.7 (Huang *et al.*, 2009a, b). For analysis of the overlap between the BA-regulated and *cop1-4*-regulated genes, DEGs co-regulated in two independent experiments using dark-grown *cop1-4* were used (Wang *et al.*, 2016; Zheng *et al.*, 2017), and for comparison with a 6 h light treatment or *det1-1* one published dataset was used (Dong *et al.*, 2014).

For real-time quantitative PCR (qPCR), four biological replicates grown for 4 days in darkness for each treatment or treated with a 6 h pulse of white light (100 µmol m^−2^ s^−1^) were collected and the real-time PCR analysis was performed as described in Cortleven *et al.* (2016). Primers used for reference genes and genes of interest are listed in Supplementary Table S1 at *JXB* online. Gene expression data were normalized against two or three different nuclear-encoded reference genes (*RPT5a, GADPH*, and/or *TAFII15*) according to (Vandesompele *et al.*, 2002) and are presented relative to the control treatment.

### Statistical analysis

Statistical analyses were performed using SAS v.9.2 (SAS Institute GmbH, Heidelberg, Germany) and Prism6 (GraphPad Software, La Jolla, CA, USA). Data were analyzed by one-way ANOVA, followed by Tukey’s post hoc test. Pairwise comparisons were made between the different treatments (control, BA, Ag, BA+Ag) for each genotype. Normality and homogeneity of variance were tested using the Shapiro–Wilk and Levene tests (Neter *et al.*, 1996). In order to meet the above-mentioned assumptions, datasets were transformed using log or square root transformation. When assumptions were not met, a non-parametric Kruskal–Wallis test was used followed by a Dunn’s multiple comparison test to perform pair-wise comparisons.

## Results

### CK promotes photomorphogenesis largely independent of ethylene

**Fig. 1.**
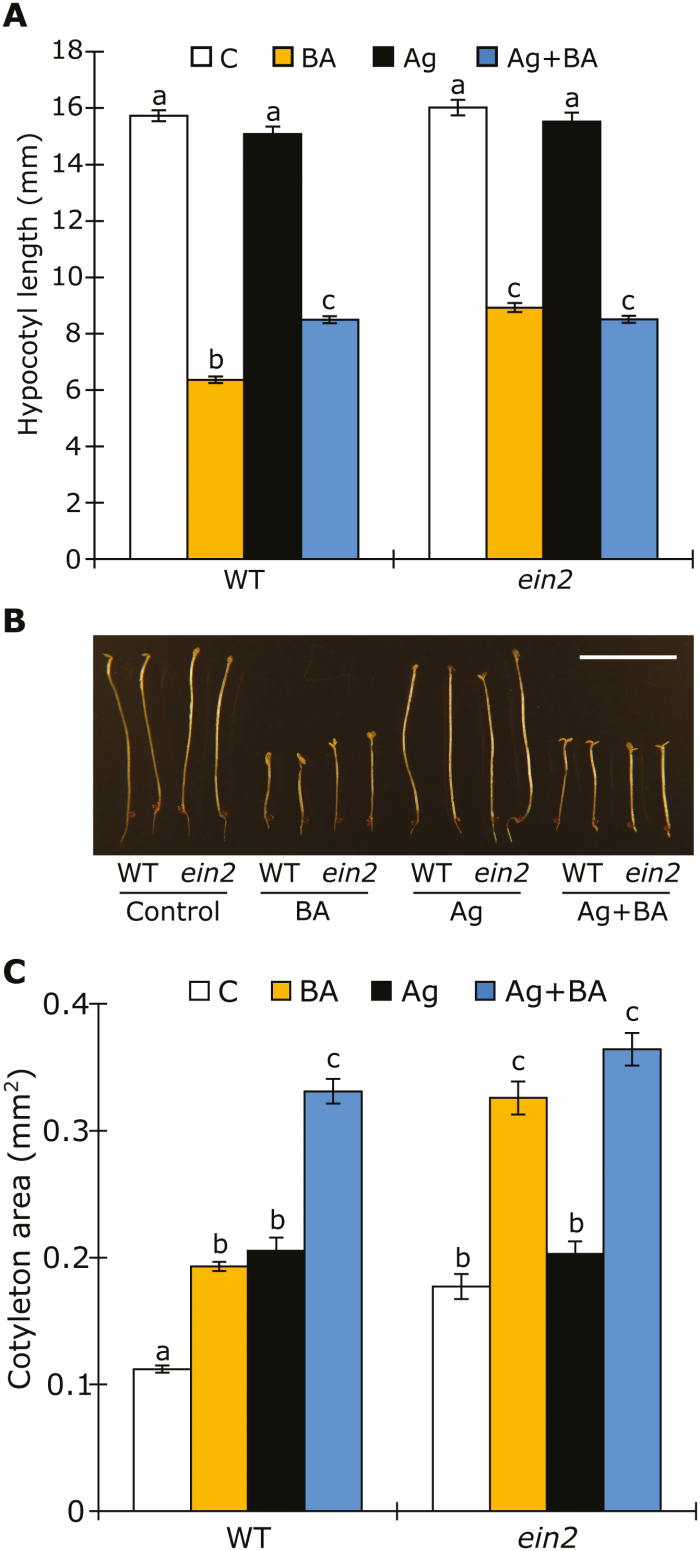
CK promotes photomorphogenesis independent of ethylene. (A) Hypocotyl length of 5-day-old etiolated WT and *ein2* seedlings growing on medium containing 10 µM AgNO_3_ (Ag) and/or 3 µM BA. C, Control. (B) Representative 5-day-old etiolated WT and *ein2* seedlings grown on medium containing 3 µM BA and 10 µM AgNO_3_. Scale bar=0 mm. (C) Area of the cotyledons of 5-day-old etiolated WT and *ein2* seedlings grown on medium containing 10 µM AgNO_3_ and/or 3 µM BA. (A, C) Error bars represent SE (*n*≥20); different letters above bars indicate statistically significant differences between experimental groups (*P*<0.05).

To examine the effects of exogenously applied BA on photomorphogenic development in relation to ethylene, we treated WT and ethylene-resistant *ein2-1* seedlings with 3 µM BA with or without the addition of AgNO_3_ as an inhibitor of ethylene signaling. As previously shown (Cary *et al.*, 1995), BA-treated seedlings grown in darkness had a short hypocotyl and exaggerated apical hook, reminiscent of the ethylene triple response (Fig. 1A, B). Strikingly, however, BA treatment of the *ein2-1* mutant also resulted in a strong inhibition of hypocotyl elongation. Likewise, BA-treated WT plants grown on media containing AgNO_3_ or aminoethoxyvinylglycine (AVG), known inhibitors of ethylene signaling and biosynthesis, respectively, also caused a strong reduction of hypocotyl elongation, suggesting the activation of an ethylene-independent pathway (Fig. 1A, B; Supplementary Fig. S1B). The effectiveness of the AgNO_3_ treatment was confirmed by its ability to completely inhibit a seedling response to 10 µM ACC in darkness (Supplementary Fig. S1A). Besides its ethylene-independent effect on hypocotyl elongation, BA treatment of *ein2-1* also resulted in opening of the cotyledons and a marked increase in cotyledon size (Fig. 1B, C). In this response, ethylene and CK appear to work antagonistically. Both BA treatment or the inhibition of ethylene signaling by AgNO_3_ alone resulted in a slight increase in the cotyledon area in both WT and *ein2-1* seedlings. However, treatment with BA in addition to AgNO_3_ (or BA treatment of the *ein2-1* mutant) resulted in further expansion (Fig. 1C).

From these data, we conclude that CK, in addition to activating ethylene signaling and biosynthesis, induces photomorphogenic development in dark-grown seedlings (characterized by a short hypocotyl and open/expanded cotyledons) largely independent of ethylene.

### CK treatment causes a transcriptional response similar to that of light treatment

**Fig. 2.**
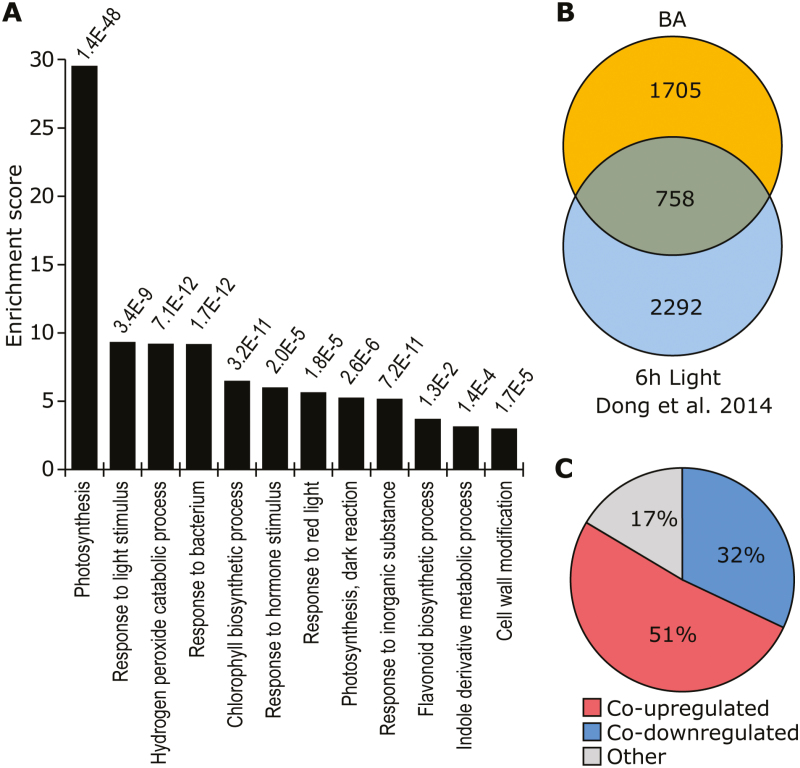
The transcriptional response to CK shows a significant overlap with the response to light treatment. (A) Gene Ontology analysis of CK-regulated genes. (B) Venn diagram showing the number of overlapping light- and CK-regulated genes. (C) Percentage of genes co-regulated by light and CK treatment.

To investigate the ethylene-independent transcriptional response to BA, RNA-Seq was performed on 4-day-old etiolated seedlings grown on medium containing 10 µM AgNO_3_ and 3 µM BA, and on 10 µM AgNO_3_-treated control seedlings. This resulted in the identification of 2463 DEGs that were significantly up-regulated (1140) or down-regulated (1323) in response to 3 µM BA (Supplementary Table S2). First, we analyzed CK metabolism and signaling genes in the RNA-Seq dataset and found that most *CKX* and A-type *ARR* genes were strongly up-regulated, consistent with the BA treatment (Supplementary Fig. S2A). Interestingly, GO term analysis of the full dataset revealed a set of GO terms (Fig. 2A), many of which were related to activated light-signaling pathways (photosynthesis, response to light stimulus, response to red light, flavonoid biosynthesis, and cell wall modification). Since many GO terms were related to light signaling, we further compared our dataset to a previously published dataset of transcripts differentially expressed in response to a 6 h light treatment (Dong *et al.*, 2014), which resulted in a significant overlap of 758 genes (*P*<0.0001, Fisher’s exact test) between light-regulated and CK-regulated genes (Fig. 2B). Of these genes, 83% were co-regulated (51% co-upregulated, 32% co-downregulated) in response to BA or light (Fig. 2C). Furthermore, GO analysis of the overlap suggested that both BA and light treatment result in the up-regulation of genes related to photosynthesis, response to light stimulus, photosynthetic electron transport chain, chlorophyll biosynthetic process, and response to red light, while down-regulated genes are related to cell wall modification and the hydrogen peroxide catabolic process (Supplementary Fig. S2B–D). As many GO terms were related to photosynthesis and light perception, and there was a large overlap with published RNA-Seq data investigating the transcriptional response to light treatment, we conclude that CK treatment of dark-grown seedlings with blocked ethylene signaling results in transcriptional changes that are partly similar to those associated with light treatment.

**Fig. 3.**
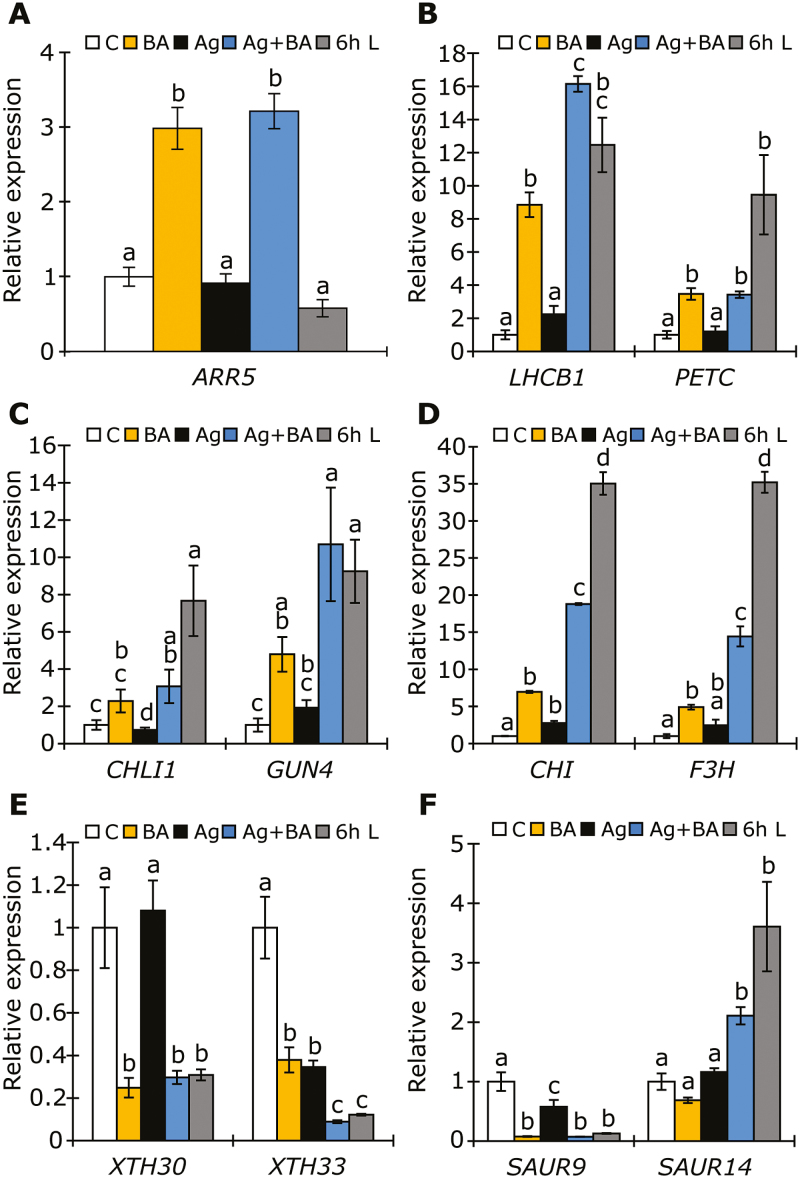
Effect of CK on transcript levels of functionally related genes. Transcript analysis by qPCR of (A) *ARR5*, (B) genes involved in chloroplast development, (C) chlorophyll biosynthesis, (D) anthocyanin biosynthesis, (E) cell wall elongation, and (F) response to auxin in 4-day-old etiolated WT seedlings grown on medium containing 3 µM BA and/or 10 µM AgNO_3_ (Ag). C, Control. Seedlings were grown in continuous darkness or received white light for 6 h prior to harvest. The transcript levels of WT in the control condition were set to 1 (*n*=4). *ACTIN2, GAPDH, RPT5*, and *TAFII15* were used as reference genes. Different letters above bars indicate statistically significant differences between experimental groups (*P*<0.05).

To confirm our RNA-Seq data and the independence of CK action from ethylene, as well as the similarity of responses to those resulting from light treatment, we performed real-time qPCR on selected targets from the BA/6 h light RNA-Seq data overlap (Fig. 2B), looking at the effects of CK treatment with and without AgNO_3_ in darkness, and the effect of a 6 h white light treatment. Total RNA was extracted from 4-day-old etiolated WT plants grown in darkness on medium containing 3 µM BA with or without 10 µM AgNO_3_, and from plants grown on control medium with or without exposure to a 6 h white light treatment. Based on the GO term analysis, transcript levels of chloroplast-related genes (*LHCB1*, *PETC*), chlorophyll biosynthesis genes (*CHLI1*, *GUN4*), anthocyanin biosynthesis genes (*CHS*, *F3H*), and genes involved in cell wall elongation (*XTH30*, *XTH33*) were measured (Fig. 3). The up-regulation of the expression of the A-type ARR *ARR5*, which is a frequently used marker gene for CK induction, indicates that the BA treatment resulted in a strong CK response (Fig. 3A). In most cases, inhibition of ethylene signaling by AgNO_3_ did not change the transcriptional response to CK in the investigated genes, suggesting that the BA-dependent induction of these genes is ethylene independent. Transcript levels of genes involved in chloroplast function (Fig. 3B, C) and anthocyanin biosynthesis (Fig. 3D) were strongly up-regulated upon both BA treatment and light treatment. Genes involved in cell wall elongation (Fig. 3E) displayed a strongly reduced level of transcripts after treatment with BA or light, consistent with the observed inhibition of hypocotyl elongation (Fig. 1). Lastly, we also analyzed *SAUR9* and *SAUR14*, which are predominantly expressed in the hypocotyl and cotyledons, respectively (Sun *et al.*, 2016). We observed that while *SAUR9* is inhibited by both BA and light, *SAUR14* is up-regulated (when ethylene signaling is inhibited), consistent with the inhibition of hypocotyl elongation and cotyledon expansion observed under these conditions (Figs 3F and 1C). While we initially compared the response to BA and light using our RNA-Seq data and that of others (Dong *et al.*, 2014) (Fig. 2), the consistent co-regulation of BA and light treatment observed by qPCR in selected targets (Fig. 3) confirms the predictability of the RNA-Seq comparison made.

Thus, we conclude that, similar to the phenotypic response (Fig. 1), the effects of CK (BA treatment) resemble those of light treatment at the transcriptional level, independent of ethylene.

### Etiolated CK-signaling mutants show a reduced photomorphogenic response to CK

**Fig. 4.**
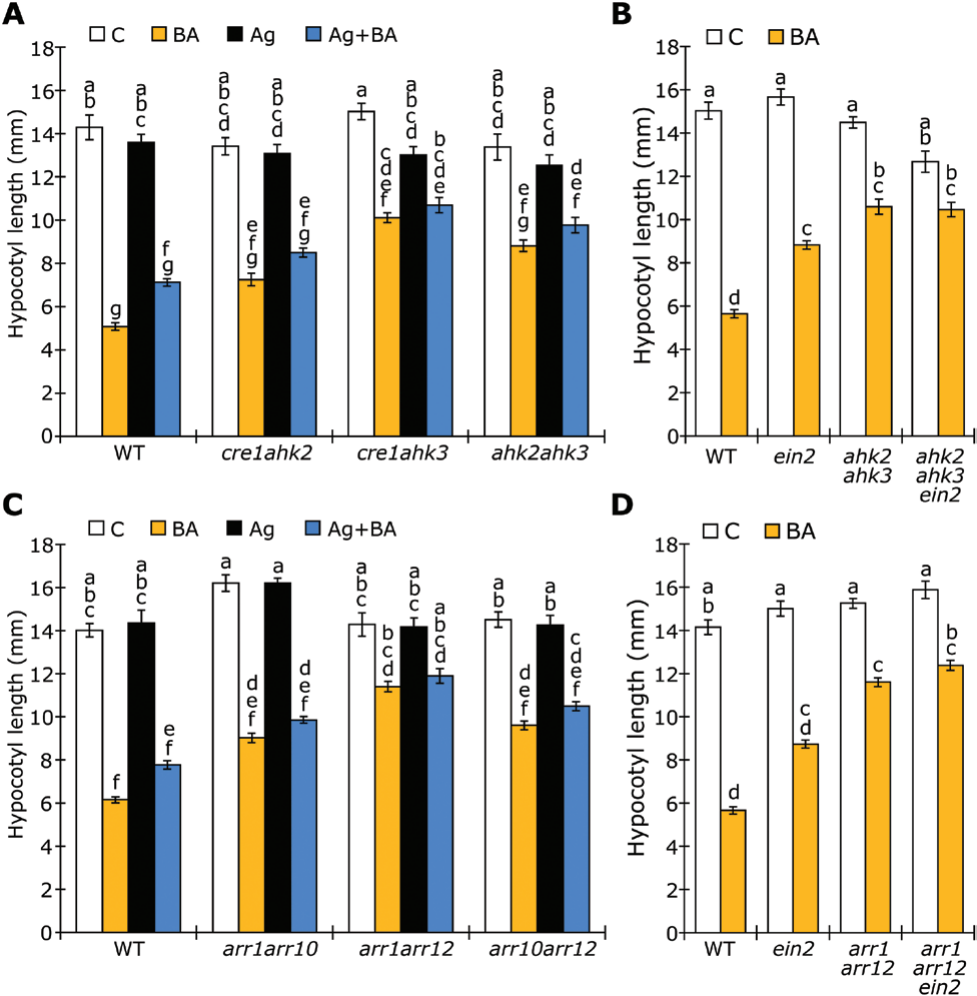
The CK receptor AHK3 and the B-type response regulators ARR1 and ARR12 are required for CK-mediated de-etiolation. Hypocotyl length of (A) 5-day-old etiolated receptor double mutants (WT, *cre1 ahk2*, *cre1 ahk3*, *ahk2 ahk3*), (B) B-type response regulator double mutants (WT, *arr1 arr10*, *arr1 arr12*, *arr10 arr12*), (C) ethylene-insensitive mutants (*ein2*) in combination with *ahk2 ahk3*, and (D) *ein2* mutants in the *arr1 arr12* background, growing on medium containing 10 µM AgNO_3_ (Ag) and/or 3 µM BA. C, Control. Error bars represent SE (*n*≥18); different letters above bars indicate statistically significant differences between experimental groups (*P*<0.05).

Although all three CK receptors and some B-type ARRs (ARR1, ARR10, and ARR12) have previously been shown to be important for the CK-dependent inhibition of hypocotyl elongation in the dark, these experiments were conducted without the inhibition of ethylene signaling (Riefler *et al.*, 2006; Argyros *et al.*, 2008). Therefore, in order to determine which CK receptor is responsible for the ethylene-independent response to BA in darkness, we analyzed *ahk2*, *ahk3*, and *cre1* single and double mutants after growth in darkness under our experimental conditions for 5 days (Fig 4A; Supplementary Fig. S3A). Analysis of the single receptor mutants revealed BA responses similar to those of the WT, regardless of the addition of AgNO_3_ (Supplementary Fig. S3A). However, analysis of the double mutants revealed a reduced response of the *cre1 ahk3* and *ahk2 ahk3* mutants, suggesting that AHK3 in combination with AHK2 or CRE1 plays a dominant role in the ethylene-independent BA response (Fig. 4A).

Similarly, although the B-type ARR mutants *arr1* and *arr12* might show a slightly reduced response to BA when not treated with AgNO_3_, the ethylene-independent response appeared largely similar to that of the WT in the *arr1*, *arr10*, and *arr12* single mutants (Supplementary Fig. S3B). To account for possible redundant functions, we further analyzed the double mutants and found that the *arr1 arr12* mutant showed reduced responsiveness to CK treatment, independently of ethylene signaling (Fig. 4C). As the *arr1 arr12* mutant exhibited reduced CK sensitivity, we performed a dose–response curve with increasing BA concentrations. While a strong gradual inhibition of hypocotyl elongation with increasing BA concentrations on media supplemented with 10 µm AgNO_3_ was observed in the WT, this inhibition was much weaker in the *arr1 arr12* mutant (Supplementary Fig. S3C). Lastly, to rule out any unknown effects of AgNO_3_ in these experiments, we generated the *ahk2 ahk3 ein2* and *arr1 arr12 ein2* triple mutants to compare their BA responsiveness to that of the *ahk2 ahk3* and *arr1 arr12* double mutants. We found that the introgression of *ein2* had no effect on the response to BA in the double mutants (Fig. 4B, D). Together, these results indicate that the receptor AHK3, in combination with either AHK2 or CRE1, and the B-type response regulators ARR1 and ARR12 play a prominent role in the regulation of hypocotyl elongation in response to CK.

**Fig. 5.**
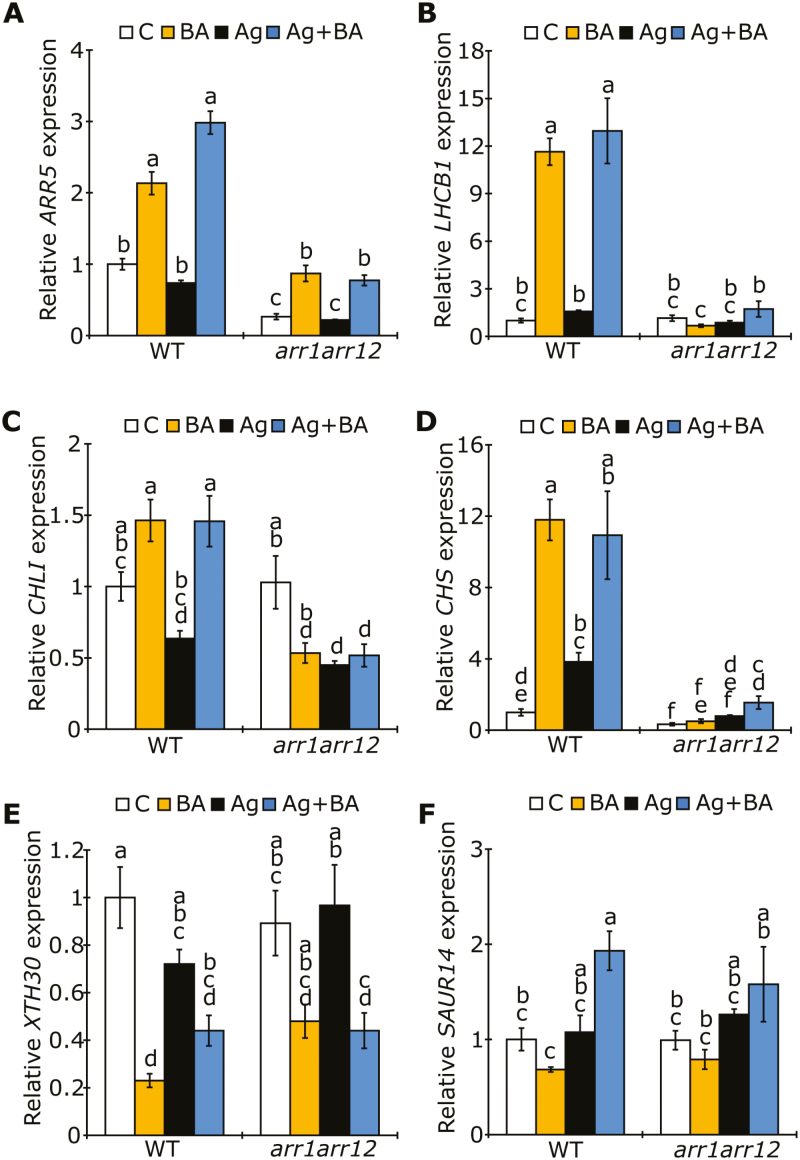
Light-regulated genes are less responsive to CK in the *arr1 arr12* mutant. Transcript analysis by qPCR of (A) *ARR5*, (B) genes involved in chloroplast development, (C) chlorophyll biosynthesis, (D) anthocyanin biosynthesis, (E) cell wall elongation, (F) and response to auxin, in 4-day-old etiolated WT and *arr1 arr12* seedlings grown on medium containing 3 µM BA and/or 10 µM AgNO_3_ C, control. The transcript level of WT in the control condition was set to 1 (*n*=4). *GAPDH*, *RPT5*, and *TAFII15* were used as reference genes. Different letters above bars indicate statistically significant differences between experimental groups (*P*<0.05).

To see whether ARR1 and ARR12 are also important for the transcriptional response to BA, transcript levels of marker genes (see also Fig. 3) were measured in the *arr1 arr12* mutants (Fig. 5). The transcript level of the CK-responsive *ARR5* gene was reduced in the *arr1 arr12* mutant in comparison to the WT; however, there was still a slight induction of expression by BA treatment in comparison to control conditions (Fig. 5A). This indicates that ARR1 and ARR12 act redundantly with other B-type ARRs to induce the expression of *ARR5*, which is consistent with previous reports (Mason *et al.*, 2005). Transcript levels of genes involved in chloroplast function (*LHCB1*, *CHLI*) were strongly reduced in *arr1 arr12* mutants in response to BA treatment relative to expression in the WT (Fig. 5B, C). *SAUR14* and *CHS* were also less responsive to BA in *arr1 arr12* mutants relative to WT, indicating that for these genes, gene expression is mediated through ARR1 and ARR12. In line with our previous results (Fig. 3), the BA-dependent regulation of these light-responsive genes is largely independent on ethylene signaling, and this finding suggests that the CK-dependent regulation of the investigated genes is mediated through ARR1 and ARR12.

### The photomorphogenic mutants *cop1, det1*, and *cin4* are CK insensitive

**Fig. 6.**
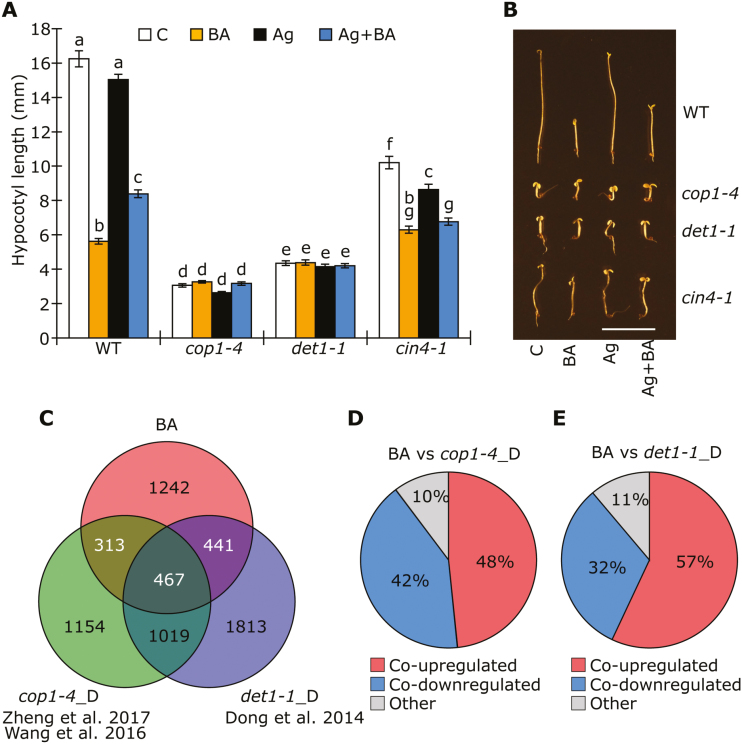
The *cop1, det1*, and *cin4* mutants are insensitive to CK in darkness. (A) Hypocotyl length of 5-day-old etiolated mutant seedlings growing on medium containing 10 µM AgNO_3_ (Ag) and/or 3 µM BA. C, Control. Error bars represent SE (*n*≥21); different letters above bars indicate statistically significant differences between experimental groups (*P*<0.05). (B) Representative 5-day-old etiolated WT, *cop1-4*, *det1-1*, and *cin4-1* seedlings grown on medium containing 3 µM BA in combination with 10 µM AgNO_3_. Scale bar=10 mm. (C) Venn diagram showing the number of overlapping regulated genes in WT treated with BA and in both *cop1-4* and *det1-1* mutants. (D) Percentage of co-regulated genes in WT treated with CK and in *cop1-4* mutants. (E) Percentage of co-regulated genes in WT treated with CK and in *det1-1* mutants.

Our results suggest that BA treatment of dark-grown seedlings results in a reduction of hypocotyl length and the expansion of cotyledons, as well as a transcriptional response partly similar to that achieved by light treatment (Fig. 1). In order to identify a possible point of convergence between the CK and light-induced pathways, several light-signaling mutants were tested for altered responses to CK treatment in darkness. As HY5 is a a major positive regulator of photomorphogenesis and shown to accumulate after BA treatment (Vandenbussche *et al.*, 2007), the effect of CK on hypocotyl elongation was investigated first in *hy5* (Supplementary Fig. S4) and *hy5 hyh* (results not shown) mutants. Both mutants responded to BA treatment in a similar manner as the WT, suggesting that HY5 and its close homolog HYH are not responsible for the CK-induced inhibition of hypocotyl elongation. Next, we investigated hypocotyl elongation in response to BA in the photomorphogenic mutants *cop1*, *det1*, and *cin4/cop10*. Unlike in the WT, the reduction in hypocotyl elongation upon BA treatment was almost completely lost in these mutants, which already had a markedly shortened hypocotyl under control conditions (Fig. 6A). To investigate whether the short photomorphogenic hypocotyl of *cop1* was the reason for the non-responsiveness to BA, the *hy5* mutant was crossed with the *cop1* mutant, which partially rescued the short-hypocotyl phenotype of *cop1* under control conditions. Although significantly longer than *cop1-4* when grown in darkness, the *hy5 cop1-4* double mutant was largely CK insensitive, consistent with a role for COP1 in the ethylene-independent CK regulation of hypocotyl elongation (Supplementary Fig. S4). Together, these results indicate that a group of negative regulators of photomorphogenesis are necessary for the ethylene-independent CK response resulting in inhibition of hypocotyl elongation in the dark.

On the basis of the above-described phenotypic observations, we expected that the *cop1*, *det1*, and *cin4* mutants would also be largely insensitive at the transcriptional level to BA treatment in the dark. Furthermore, as these mutants phenotypically resemble BA+AgNO_3_-treated WT seedlings (Fig. 6B), there might be a transcriptional overlap between the mutants and BA-treated WT seedlings. To test this hypothesis, we used two RNA-Seq datasets comparing *cop1-4* to the WT grown in darkness from the literature (Wang *et al.*, 2016; Zheng *et al.*, 2017). By comparing these two datasets, we found 2953 genes consistently 2-fold deregulated in *cop1-4*. Furthermore, we examined a list of *det1-1* regulated genes in seedlings grown in darkness (Dong *et al.*, 2014). By comparing these datasets with the BA-regulated genes, we found significant overlaps between both *cop1-4*/BA (780 genes) and *det1-1*/BA (908 genes) (Fig. 6C; Supplementary Table S3). Furthermore, 90% and 89% of these genes were co-regulated in *cop1-4* and *det1-1* relative to BA treatment, respectively, suggesting that mutation of these genes results in a partly similar response to that of WT treated with BA (Fig. 6D, E). Lastly, GO term analysis of genes co-regulated by BA and *cop1-4* or *det1-1* showed a remarkable similarity to the GO term analysis of all genes regulated by BA (Supplementary Fig. S5A, B;Fig. 2A), which indicates that COP1 and DET1 might be essential for the photomorphogenic CK response.

**Fig. 7.**
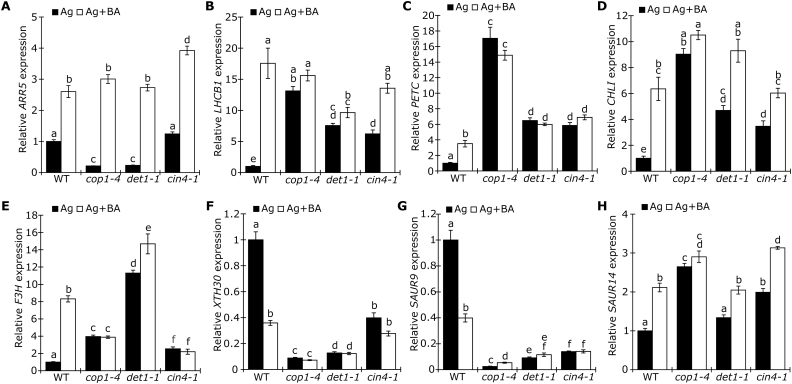
Effect of CK on transcript levels of functionally related genes in the *cop1, det1*, and *cin4* mutants. Transcript analysis by qPCR of (A) *ARR5*, (B, C) genes involved in chloroplast development, (D) chlorophyll biosynthesis, (E) anthocyanin biosynthesis, (F) cell wall elongation, and (G, H) response to auxin in 4-day-old etiolated WT, *cop1-4*, *det1-1*, and *cin4-1* seedlings grown on medium containing 10 µM AgNO_3_ with or without 3 µM BA. The transcript level of WT in the control condition was set to 1 (*n*≥4). *GAPDH, RPT5*, and *UBC21* were used as reference genes. Different letters above bars indicate statistically significant differences between experimental groups (*P*<0.05).

To provide further support for the transcriptomic analysis and to investigate the sensitivity to BA of these mutants at the transcriptional level, the ethylene-independent CK effect was analyzed based on the transcript levels of marker genes (see Fig. 3) in 4-day-old etiolated seedlings. Although it was suppressed in *cop1* and *det1* under control conditions, we confirmed that *ARR5*, a transcriptional marker for the central CK response, is still up-regulated in response to BA in *cop1*, *det1*, and *cin4* when grown on AgNO_3_, suggesting that the CK-signaling pathway is fully functional in these mutants (Fig. 7A). Transcript levels of *LHCB1*, *PETC*, *CHL1*, *F3H*, and *SAUR14* were promoted in response to BA in the WT, while *XTH30* and *SAUR9* were suppressed (Fig. 7B–H). A similar observation for transcriptional behavior was also shown in the WT after CK treatment (Fig. 3).Furthermore, in line with the suggested transcriptional overlap (Fig. 6), *cop1*, *det1*, and *cin4* mutants showed regulation under control conditions (treatment with AgNO_3_ alone) similar to BA-treated WT (AgNO_3_ + BA); these mutants have constitutively active light signaling, and these findings thus again confirmed the light-mimicking effect of CK. Importantly, in most cases, changes in basal expression and a strikingly reduced response to BA treatment was observed in the three mutants, suggesting that COP1, DET1, and CIN4 not only regulate the same genes as BA in darkness, but also are required for BA responsiveness.

Taken together and considering the phenotypic analysis of the mutants, these results suggest that COP1, DET1, and CIN4 might be important for the transcriptional response to CK.

### Light triggers a switch in CK function

**Fig. 8.**
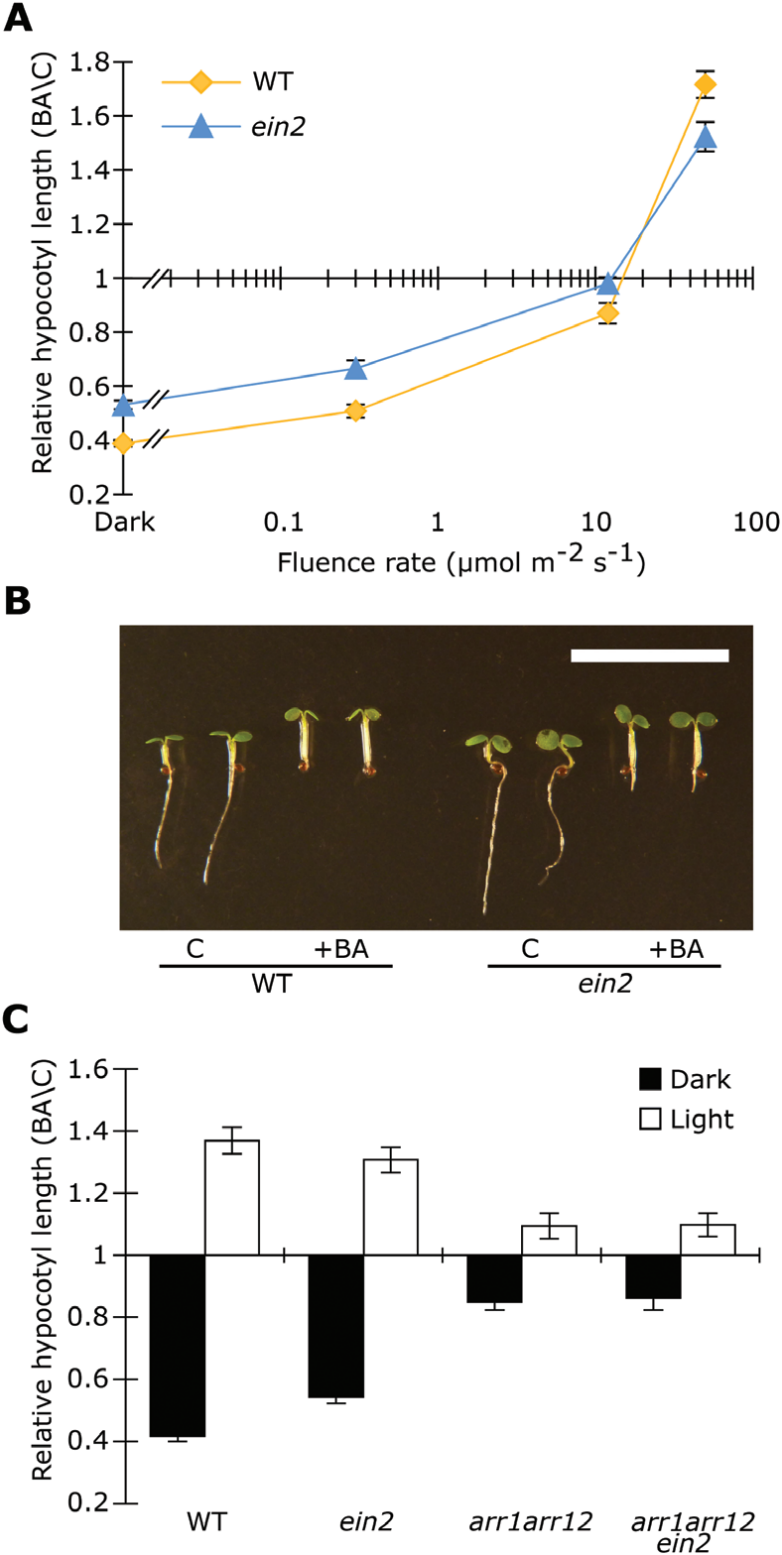
Light inhibits the CK-dependent suppression of hypocotyl elongation. (A) Relative hypocotyl length of 5-day-old WT and *ein2-1* seedlings growing on medium with or without 3 µM BA under different light fluence rates (0–50 µmol m^−2^ s^−1^). The data are presented as the ratio of BA-treated seedlings to seedlings grown under control conditions. Error bars represent SE (*n*≥24). Experiments were performed at least twice; results from one representative experiment are shown. (B) Representative 5-day-old WT and *ein2-1* seedlings grown on medium with or without 3 µM BA. Scale bar=10 mm. (C) Relative hypocotyl length of 5-day-old WT, *ein2*, *arr1arr12*, and *arr1arr12ein2* seedlings growing on medium with or without 3 µM BA in the dark or in light (50 µmol m^−2^ s^−1^). Data are presented as in (A). Error bars represent SE (*n*≥20).

As our results suggest that COP1, DET1, and CIN4 are required for CK-dependent photomorphogenesis in darkness, we hypothesized that light, which inhibits the function of these factors, would also inhibit the effects of CK on suppressing hypocotyl elongation. To test this hypothesis, we grew WT and *ein2* seedlings with or without 3 µM BA under increasing fluence rates of white light. As reported above, the addition of BA in darkness resulted in a strong inhibition of hypocotyl elongation in both the WT and the *ein2* mutant (Figs 1 and 8A). When the fluence rate of light was increased to 10 µmol m^−2^ s^−1^, the relative effect of the addition of BA decreased to a point of almost no response. This is consistent with the hypothesis that light, possibly acting through COP1/DET1/CIN4, inhibits the effects of BA (Fig. 8A). Interestingly, when we further increased the fluence rate of light to 50 µmol m^−2^ s^−1^ we observed a CK-dependent promotion of hypocotyl elongation (Fig. 8A, B). As this effect was also observed in the *ein2* mutant, albeit to a slightly lesser degree, we conclude that in light, the CK-dependent promotion of hypocotyl elongation is independent of ethylene, similar to the CK-dependent inhibition of hypocotyl elongation in darkness.

To investigate whether the promotion of hypocotyl elongation induced by CK is regulated by the same B-type ARRs as the inhibitory effects seen in darkness, WT, *ein2*, *arr1 arr12*, and *arr1 arr12 ein2* mutants were grown in darkness and under 50 µmol m^−2^ s^−1^ of white light for 5 days. Compared with the WT, the *arr1 arr12* seedlings showed a strongly reduced response in light as well as a strongly reduced response in darkness, as has been previously shown (Figs 4D, 8C; Supplementary Fig. S3C), suggesting that ARR1 and ARR12 are important regulators of both of these responses. Furthermore, as the *arr1 arr12 ein2* triple mutant behaved in a similar fashion to the *arr1 arr12* mutant in both conditions, we conclude that the CK-dependent regulation of hypocotyl elongation is largely ethylene independent, regardless of the light conditions (Fig. 8C).

In conclusion, in line with the importance of COP1/DET1/CIN4 in the CK-induced inhibition of hypocotyl elongation in darkness, the addition of light suppresses this BA-dependent phenotypic response. However, greater amounts of light surprisingly result in a switch of the response, whereby CK action changes from an inhibitor to a promoter of hypocotyl elongation.

## Discussion

**Fig. 9.**
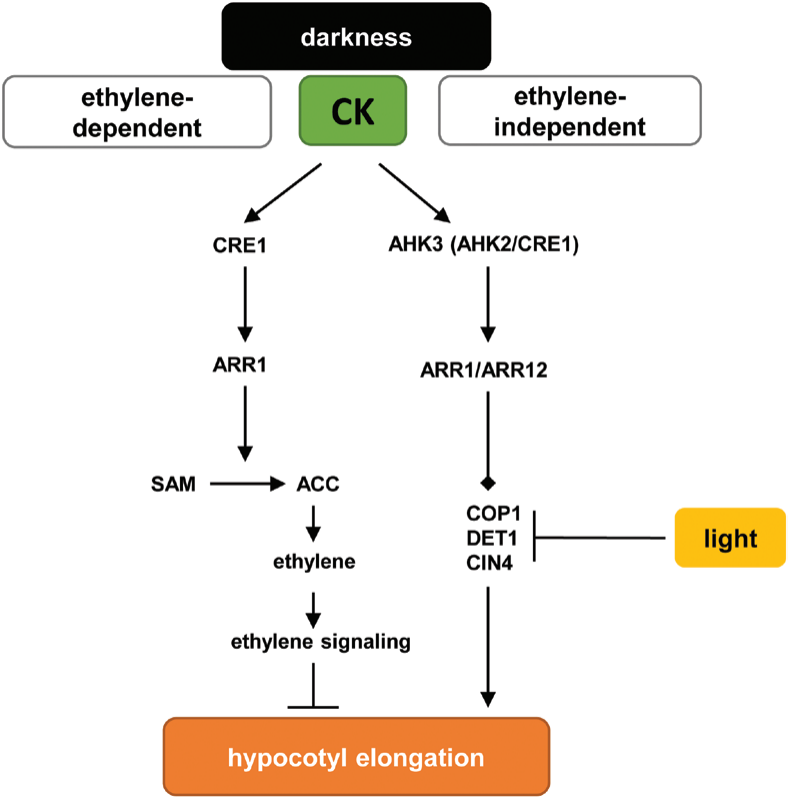
Model for the CK-dependent photomorphogenic response in etiolated Arabidopsis seedlings. Dark-grown seedlings undergo skotomorphogenesis, which is characterized by elongation of the hypocotyl, closed cotyledons, and the formation of an apical hook. In the presence of CK, a photomorphogenic response results in the inhibition of hypocotyl elongation. This response can be either ethylene dependent (Cary *et al*, 1995; Woeste *et al.*, 1999; Hansen *et al.*, 2009) or ethylene independent (as reported in this study). In the ethylene-independent signaling pathway, the CK signal is mediated through AHK3, in cooperation with either AHK2 or CRE1/AHK4, via the B-type ARRs ARR1 and ARR12 to inhibit hypocotyl elongation via the modulation of COP1/DET1/CIN4. ACC, 1-aminocyclopropane-1-carboxylic acid; SAM, *S*-adenosyl-l-methionine.

In this study, we re-examined the CK response in dark-grown seedlings by using hypocotyl elongation as a proxy for the CK response. CK promotes photomorphogenic development largely independent of ethylene signaling. The de-etiolated phenotype of CK-treated seedlings correlates with a transcriptional response resembling a light treatment or a mutation in the *COP/DET/FUS* loci. Furthermore, inhibition of the COP1/CDD module by mutation resulted in CK insensitivity with regard to both hypocotyl elongation and transcriptional regulation of marker genes, suggesting a prominent role of these factors in the pathway by which CK promotes photomorphogenesis. A model describing our findings is shown in Fig. 9. These intricate connections between the light- and CK-signaling networks provide an initial molecular framework for further investigation.

### CK promotes photomorphogenesis largely independent of ethylene signaling

Although it is known that CK promotes photomorphogenic development in darkness (Chory *et al.*, 1994), the underlying molecular networks mediating this response are still largely unknown. Moreover, it has been suggested that this photomorphogenic response to CK is mediated by increased ethylene biosynthesis (Cary *et al.*, 1995). However, the fact that high CK levels induce the opening of cotyledons and the emergence of true leaves (Chory *et al.*, 1994; Riefler *et al.*, 2006) suggests the presence of an ethylene-independent pathway by which CK induces photomorphogenic development in dark-grown seedlings.

Here, we have re-examined the role of CK and ethylene in the inhibition of hypocotyl elongation in darkness. As previously reported (Cary *et al.*, 1995), BA treatment resulted in a strong inhibition of hypocotyl elongation and a seedling morphology resembling the ethylene triple response (Fig. 1A, B). However, the inhibition of ethylene signaling by AgNO_3_, AVG, or the use of the ethylene-insensitive *ein2* mutant revealed that the majority of the CK-induced inhibition of hypocotyl elongation is largely ethylene independent (Fig. 1A, B; Supplementary Fig. S1B). Thus, these data suggest that the triple response observed when seedlings are treated with CK might mask CK-dependent photomorphogenic development, which can be overcome either by the addition of very high levels of CK (Chory *et al.*, 1994) or by inhibiting ethylene signaling (as we found in this study).

To further characterize the ethylene-independent de-etiolation response to CK, we performed RNA-Seq analysis. This identified 2463 DEGs in many biological processes, but especially related to light signaling, photosynthesis, and cell wall modifications in GO term analysis (Fig. 2A; Supplementary Fig. S2B–D). In addition, comparison of these DEGs with genes responsive to a 6 h light treatment revealed a significant overlap, indicating that CK treatment might result in the activation of light-signaling networks (Fig. 2B, C).

In conclusion, with this set of experiments we demonstrated that CK promotes de-etiolation in dark-grown seedlings largely independent of ethylene signaling, and that this response resembles a light treatment, both phenotypically and at the transcriptomic level.

### CK regulates photomorphogenic responses through the action of mainly the AHK3 receptor and ARR1 and ARR12 B-type response regulators

CK perception and signaling has been elucidated in detail in *Arabidopsis thaliana* (for review, see Kieber and Schaller, 2014). Distinct functions have been attributed to the different parts of the CK-signaling pathway. For instance, the CK receptors AHK2, AHK3, and CRE1 have been assigned a variety of developmental and physiological functions (Higuchi *et al.*, 2004; Nishimura *et al.*, 2004; Riefler *et al.*, 2006). Our results suggest that AHK3, in combination with either AHK2 or CRE1, is involved in mediating the CK signal in dark-grown seedlings (Fig. 4A). These results are in line with the finding that AHK3 is important for CK-induced photomorphogenesis (Riefler *et al.*, 2006) and with the recent discovery that a combined loss of AHK3 and AHK2 results in retarded chloroplast development during the transition from dark to light (Cortleven *et al.*, 2016). Moreover, from an evolutionary perspective, AHK2 and AHK3 are more closely related to each other than to CRE1/AHK4, and both receptors are predominantly expressed and active in shoot tissues (Ueguchi *et al.*, 2001; Higuchi *et al.*, 2004; Stolz *et al.*, 2011).

Downstream of the CK receptors, the B-type response regulators mediate CK activity. In this study, we focused on the transcription factors ARR1, ARR10, and ARR12, since these are known to be responsible for most CK-related responses (Mason *et al.*, 2005; Yokoyama *et al.*, 2007; Argyros *et al.*, 2008; Ishida *et al.*, 2008). The *arr1 arr12* mutant clearly exhibited a diminished CK response in comparison to WT, both in terms of hypocotyl elongation and at the molecular level (Fig. 4B, D, Fig. 5). This points to a redundant role for ARR1 and ARR12 in CK-mediated de-etiolation and corresponds to previous reports showing that these B-type response regulators are involved in the ethylene-dependent CK response and its effect on hypocotyl elongation during growth in the dark (Argyros *et al.*, 2008). Redundant functions for ARR1 and ARR12 have also been shown for chloroplast development (Cortleven *et al.*, 2016) and protection against high light levels (Cortleven *et al.*, 2014).

B-type response regulators act through directly binding to specific sites on the promotor region of CK-regulated genes, resulting in transcriptional responses (Rashotte *et al.*, 2003; Brenner *et al.*, 2005; Taniguchi *et al.*, 2007; Brenner and Schmülling, 2012; Bhargava *et al.*, 2013). Recently, a list of putative ARR1, ARR10, and ARR12 targets has been published (Zubo *et al.*, 2017; Xie *et al.*, 2018). We explored whether any of our investigated marker genes and components of the light-signaling pathway are among the targets of these B-type ARRs. Both COP1 and HY5 were found to be putative targets of ARR1, ARR10, and ARR12 (Xie *et al.*, 2018). Similarly, PIF3, PIF4, and PIF5 were also found to be possible targets of these B-type RRs (Zubo *et al*, 2017; Xie *et al.*, 2018). Although we did not find an altered expression level of these light-signaling components after CK treatment (Supplementary Table S2), these data clearly indicate that the CK-signaling pathway might directly interact with the transcriptional regulation in a context-dependent manner and through an as yet unknown mechanism. Only a few of the strongly regulated CK marker genes were among the putative B-type ARR targets, again indicating a certain discrepancy between the developmental stage (de-etiolation) and the conditions used in the experiments of Zubo *et al.* (2017) and Xie *et al.* (2018).

Taken together, our findings show that a functional CK-signaling pathway is essential for the ethylene-independent CK response and its effect on hypocotyl elongation during photomorphogenesis.

### The COP1/CDD module links CK and light signaling

Although CK is known to regulate many light-induced responses, including the inhibition of hypocotyl elongation, and the accumulation of chlorophyll and anthocyanin, few direct links between CK and the light-signaling networks have been described (Chory *et al.*, 1994; Das *et al.*, 2012; Cortleven *et al.*, 2016). An exception is the role of the central positive regulator of photomorphogenesis, HY5, which was shown to be required for CK-induced anthocyanin accumulation under blue light (Vandenbussche *et al.*, 2007). Moreover, HY5 protein levels accumulated in response to CK treatment in WT seedlings, while no additional accumulation was observed in the *cop1* mutant, suggesting that COP1 might play a role in mediating the role of CK in anthocyanin accumulation. Although our results rule out a role of HY5 in the response to CK in darkness, the negative regulators of photomorphogenesis, COP1, DET1, and CIN4/COP10, appear to be largely required for this response (Fig. 6; Supplementary Fig. S4). By comparing the DEGs in response to BA with genes misregulated in *cop1* and *det1*, we found that ~50% of the BA-regulated genes were also regulated by COP1 and/or DET1 (Fig. 6C–E). Importantly, we further showed that the transcriptional response to CK in the *cop1*, *det1*, and *cin4* mutants was strongly reduced (Fig. 7). As these constitutive photomorphogenic mutants are largely insensitive to CK at both the phenotypic and the transcriptional level, we conclude that the promotion of de-etiolation by CK might require a functional COP1/CDD module. This is in agreement with a study demonstrating that a mutant of the pea *COP1* homolog, *LIP1,* also shows a reduced response to isopentenyladenine. This also suggests the requirement for a functional COP1 in CK responses (Sullivan and Gray, 2000).

The ability of COP1 to target positive regulators of photomorphogenesis for degradation is inhibited by light-dependent nuclear exclusion (Pacín *et al.*, 2014). Interestingly, under light conditions, ethylene inhibition of photomorphogenesis as a result of decreased HY5 stability is achieved through the promotion of COP1 nuclear localization (Yu *et al.*, 2013). Although appealing, a mechanism by which CK regulates COP1 cellular localization appears to be unlikely, as it has previously been shown that COP1-GUS is unaffected by CK treatment in etiolated seedlings (von Arnim *et al.*, 1997). Alternatively, COP1 and the CDD complex could be either transcriptionally down-regulated or directly inhibited by CK (via B-type ARRs) in the nucleus. Although transcriptional regulation is not supported by the results of our RNA-Seq experiment (Supplementary Table S2), in these scenarios CK treatment would result in the accumulation of photomorphogenesis-promoting COP1 targets other than HY5, as our data suggest that HY5 is not required for the CK-induced inhibition of hypocotyl elongation (Supplementary Fig. S4). Lastly, we cannot rule out the possibility that the CK-signaling pathway interferes with some of the non-canonical functions of these factors. For example, DET1 has been reported to both act as a transcriptional repressor and be involved in chromatin remodeling (Benvenuto *et al.*, 2002; Lau *et al.*, 2011). Taken together, we have clearly shown that a functional COP1/CDD module is necessary for the CK-mediated light-induced response in terms of hypocotyl elongation. However, the mechanism by which this is regulated is as yet unknown.

### Light-dependent switch of CK action resembles ethylene signaling

As our data demonstrated that the COP1/CDD module is required for the ethylene-independent CK response, we hypothesized that light, which inhibits the action of COP1/CDD, would also desensitize the response to CK (Osterlund *et al.*, 2000; Yanagawa *et al.*, 2004; Pacín *et al.*, 2014). In support of this hypothesis, we demonstrated that light levels up to 10 µmol m^−2^s^−1^ resulted in a gradual decrease of relative CK sensitivity (Fig. 8A). At higher levels of light, however, a striking switch of the CK response was observed whereby BA treatment resulted in elongation of the hypocotyl (Fig. 8). A similar observation concerning the effect of CK on hypocotyl elongation in light has been reported (Smets *et al.*, 2005). However, in contrast to our observations, that study showed that the hypocotyl elongation was completely dependent on the presence of the ethylene inhibitor AgNO_3_ in the medium. Regardless, this observed light-dependent switch appears analogous to the ethylene-dependent regulation of hypocotyl elongation, where ethylene inhibits elongation in the dark and promotes elongation in light at fluence rates exceeding 10 µmol m^−2^s^−1^ (Zhong *et al.*, 2012). However, the fact that the *ein2* mutant showed only a partially reduced response relative to the WT suggests that the effect of CK in light is largely independent of ethylene signaling (Fig. 8). Intriguingly, recent studies have described the direct interaction and activation of the *TAA1* promoter by ARR1, ARR10, and ARR12 (Reyes-Olalde *et al.*, 2017; Yan *et al.*, 2017). As reduced *TAA1* transcript levels in the *arr1 arr10 arr12* mutant were evident only after a light treatment, this suggests that these B-type ARRs might promote auxin-driven cell elongation specifically in light-grown seedlings. In addition, CK treatment indirectly promotes the transcription of *YUC8*, a rate-limiting enzyme in auxin biosynthesis, through ARR1/ARR12-dependent transcriptional regulation of *PIF4* (Di *et al.*, 2016). Thus, it is possible that the influence of CK on hypocotyl elongation might switch from a COP1/CDD-dominated pathway in the dark to being driven by auxin under light.

## Supplementary data

Supplementary data are available at *JXB* online.

**Fig. S1.** Ethylene-independent CK responses resemble the effect of light during photomorphogenesis.

**Fig. S2.** CK treatment causes a strong transcriptional regulation.

**Fig. S3.** Effect of CK on hypocotyl elongation in CK receptor, B-type response regulator, and ethylene-insensitive mutants.

**Fig. S4.** CK response is independent of HY5 but dependent on COP1.

**Fig. S5.** The transcriptional response to CK shows significant overlap with the transcriptional response in *cop1* and *det1* mutants.

**Table S1.** Sequences of primers used in this study.

**Table S2.** List of DEGs regulated by BA treatment.

**Table S3.** List of DEGs regulated by BA and overlap with *cop1*-, *det1*-, and light-regulated DEGs.

## Data deposition

RNA-Seq data are deposited in the NCBI Gene Expression Omnibus under GEO Series accession number GSE108912 (https://www.ncbi.nlm.nih.gov/geo/query/acc.cgi?acc=GSE108912).

## Supplementary Material

Supplementary Figures S1-S5 and Table S1

Supplementary Table S2

Supplementary Table S3
