# Supplementary material for: Ethylene-independent promotion of photomorphogenesis in the dark by cytokinin requires COP1 and the CDD complex
Source: J Exp Bot. 2018 Sep 29;70(1):165–78. doi: 10.1093/jxb/ery344 (PMC6305196; doi:10.1093/jxb/ery344)
Supplement: Supplementary Figures S1-S5 and Table S1 [file ery344_suppl_supplementary_figures_s1-s5_table_s1.pdf]

Supplementary data

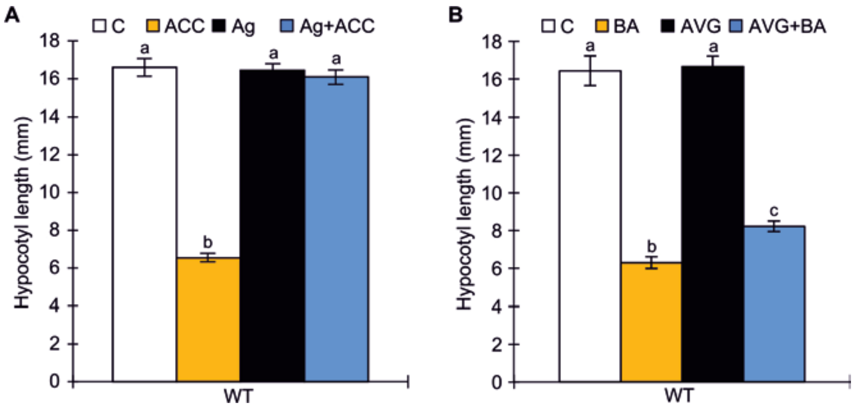

Figure S1. Ethylene-independent CK responses resemble the effect of light during photomorphogenesis.

(A-B) Hypocotyl length of 5-days-old etiolated WT seedlings growing on medium containing 10 µM $AgNO_3$ and 5 µM ACC (A) or 3 µM BA and 2 µM AVG (B). Error bars represent SE (n ≥ 28), letters represent statistically significant differences between experimental groups (0,05 > p-value).

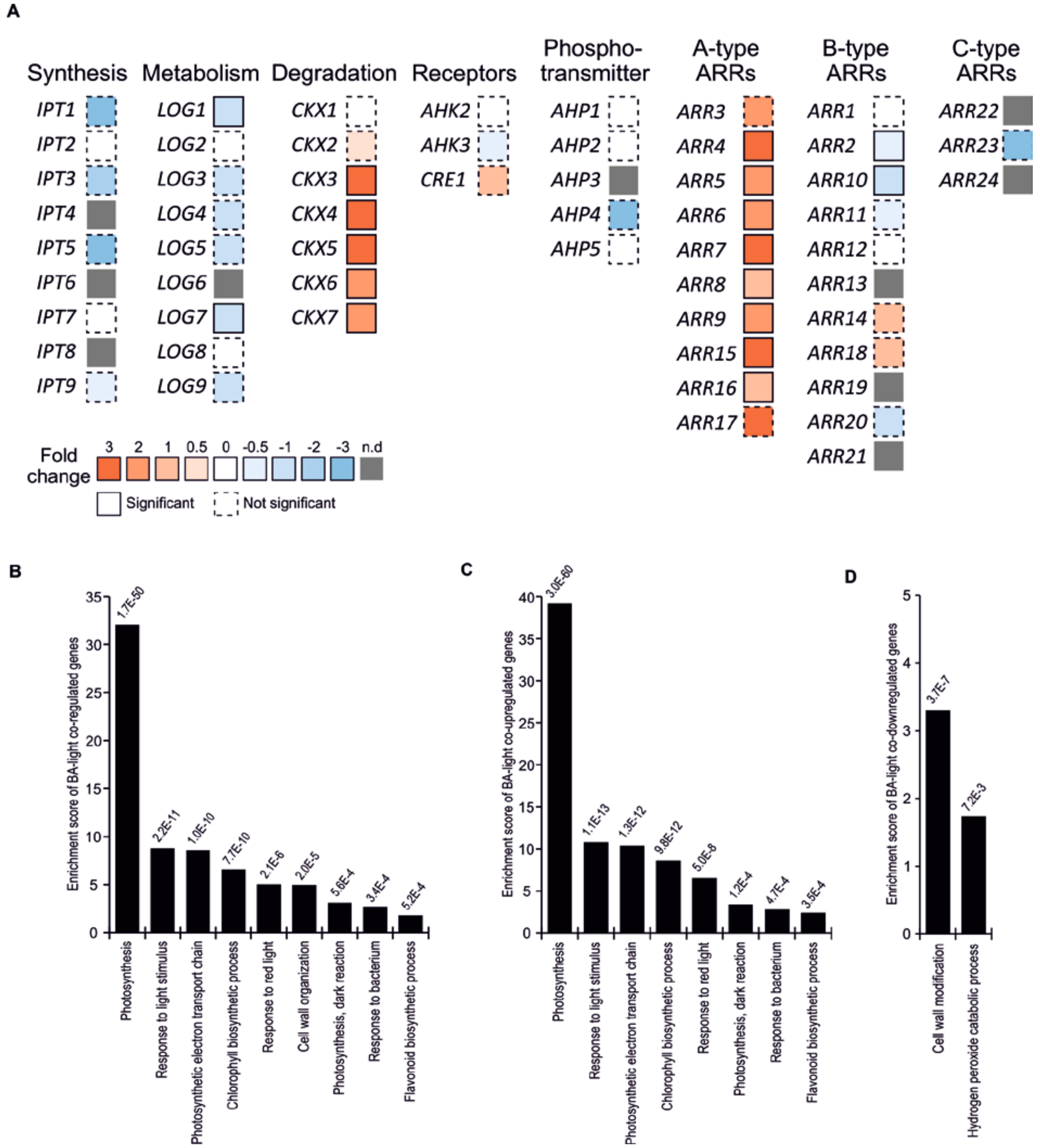

Figure S2. CK treatment causes a strong transcriptional regulation.

(A) Heat map of CK biosynthesis and signaling genes. Blue to red colors indicate fold changes in 4-days-old etiolated seedlings after CK treatment. (B-D) Gene ontology analysis of light and CK coregulated (B), co-upregulated (C) and co-downregulated (D) genes.

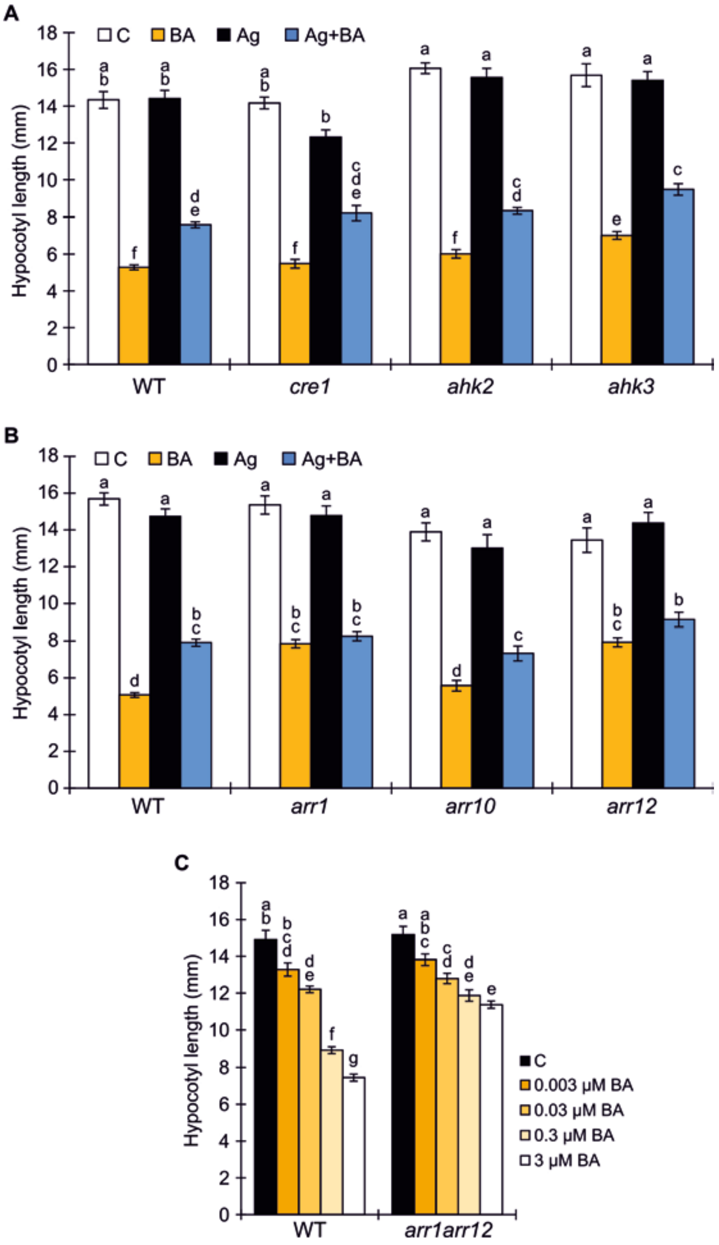

Figure S3. Effect of CK on hypocotyl elongation in CK receptor, B-type response regulator and ethylene-insensitive mutants.

Hypocotyl length of 5-days-old etiolated receptor single mutants (WT, *cre1, ahk2, ahk3*) (A) and B-type response regulator double mutants (WT, *arr1, arr10,* arr12) (B), growing on medium containing 10 µM $AgNO_3$ and/or 3 µM BA. (C) Hypocotyl length of 5-days-old etiolated seedlings grown on medium containing 10 µM $AgNO_3$ and 0,003; 0,03; 0,3 or 3 µM BA. Error bars represent SE ($n \geq 11$). Letters represent statistically significant differences between experimental groups (p-value < 0.05).

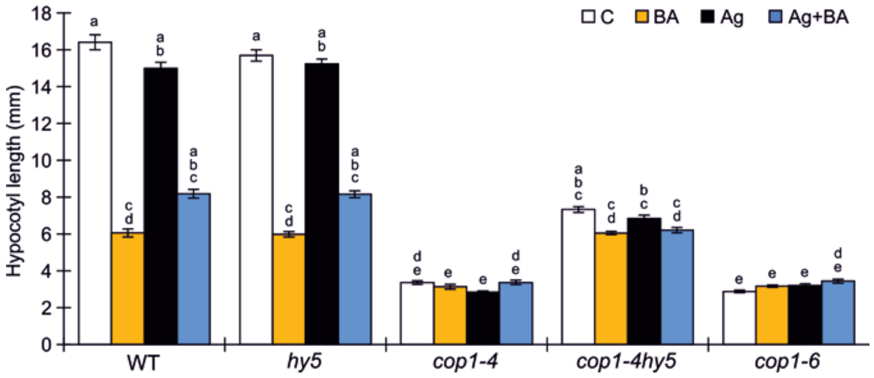

Figure S4: CK response is independent of HY5 but dependent of COP1.

Hypocotyl length of 5-days-old etiolated seedlings (WT, *hy5, cop1, cop1 hy5*) growing on medium containing 10 µM $AgNO_3$ and/or 3 µM BA; Error bars represent SE ($n \geq 22$). Letters represent statistically significant differences between experimental groups (p-value < 0.05).

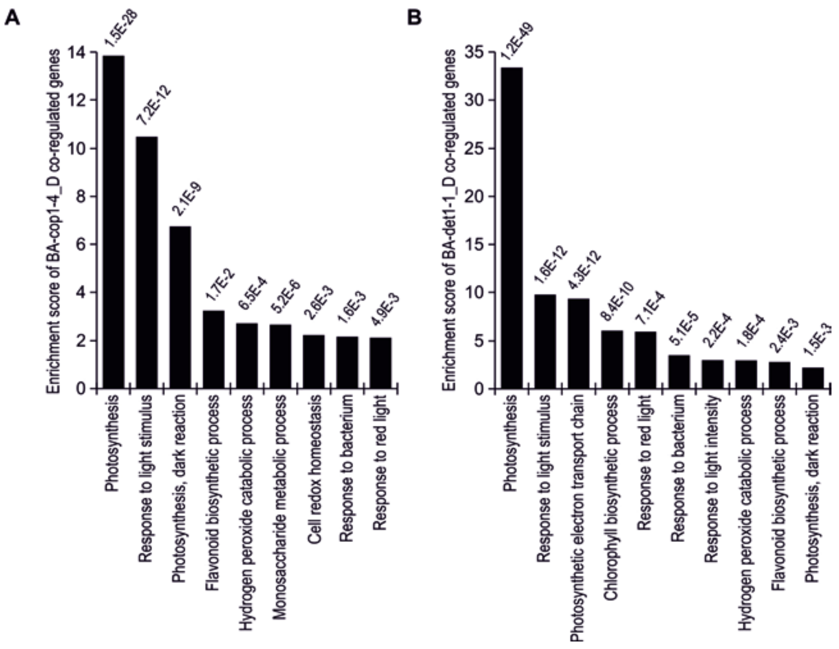

Figure S5: The transcriptional response to CK shows significant overlap with the transcriptional response in *cop1* and *det1* mutants.

(A) Gene ontology analysis of CK and *cop1-4* coregulated and (B) of CK and *det1-1* co-regulated genes.

Table S1. Sequences of primers used in this study.

Reference Genes

Gene	ATG No.	Forward Primer 5´-3´	Reverse Primer 5´-3´
ACTIN	AT3G18780	CTTGCACCAAGCAGCATGAA	CCGATCCAGACACTGTACTTCCTT
GADPH	AT3G04120	AGGTGCTTCCAGCTCTTAACG	TGCCTTCGGATTCCTCCTTG
RPT5a	AT3G05530	GGTACAAAGCGTTTCGACAGTG	TTGCTCTCGCTTCTTCGGTTG
TAFII15	AT1G17440	GAATCACGGCCAACAATC	ACTCTTAGCCAAGTAGTGCTCC
UBC21	AT5G25760	CTCTTAACTGCGACTCAGGGAATC	TGCCATTGAATTGAACCCTCTCAC

Genes of Interest

Gene	ATG No.	Forward Primer 5´-3´	Reverse Primer 5´-3´
ARR5	AT3G48100	GCCGAAAGAATCAGGACA	CTACTCGCAGCTAAAACGC
CHI	AT3G55120	CCTTTTCGTCCTTGTTCTTCATC	CCTTTTCGTCCTTGTTCTTCATC
CHLI1	AT4G18480	TTCTGGTTGGAATACGGTTGA	CTTGTCCTGCTCGGTTTTGT
F3H	AT3G51240	CAGGGACGAAGATGAACGGC	AAGCAAAGAAGTCACGAGCG
GUN4	AT3G59400	CTGCTCTGCTTCTTCCACCT	TCTCCTCGTCGGCTTGTCT
LHCb1	AT1G29910	GTGACAATGGCTTGAACGAA	GGCTACAGAGTCGCAGGAAA
PETC	AT4G03280	TTCCAGCAGACAGAGTTCCAG	ACATCGTTTCCAAGGGCATCC
SAUR14	AT4G38840	CTCCGACAAGCCAAACTGTTG	TCACTGCAAGGGATTGTGAGG
SAUR9	AT4G36110	GCACGAGATGGGTCTCACTATC	CCGAGTCTGGAGTGTGACAATG
XTH30	AT1G32170	AAAGCCGTGGAGGTTTCAGAC	CTGCTCCCATTGCATCGTTTC
XTH33	AT1G10550	GATCCAACGCAAGCCTTTCAC	CAGCAACGGAAACCACGAAAG
